# A Rare Case of Primary Infiltrating Neuroendocrine Carcinoma of the Breast

**DOI:** 10.5812/iranjradiol.8517

**Published:** 2012-11-20

**Authors:** Ouzreiah Nawawi, Keat Ying Goh, Kartini Rahmat

**Affiliations:** 1 Department of Biomedical Imaging, University of Malaya Medical Center, Kuala Lumpur, Malaysia; 2 University Malaya Research Imaging Center (UMRIC), Faculty of Medicine, University Malaya, Kuala Lumpur, Malaysia

**Keywords:** Carcinoma, Neuroendocrine, Breast, Ultrasonography

## Abstract

Primary neuroendocrine carcinoma of the breast is a very rare malignant tumor. There are not many cases reported in the English literature since it was first documented in 1983. Reports on the imaging features, in particular the ultrasonographic features of this rare tumor are scarce. Herein, we report a case of aggressive primary infiltrating neuroendocrine carcinoma of the breast, masquerading as an inflammatory breast condition in a 22-year-old young lady, perhaps the youngest case ever reported in the English literature. We discuss the imaging features and highlight the Doppler ultrasonographic findings of this rare breast carcinoma. This is the first documentation on Doppler ultrasonographic findings of primary neuroendocrine carcinoma of the breast in the literature.

## 1. Introduction

Primary neuroendocrine carcinoma of the breast is one of the uncommon variants of extra pulmonary neuroendocrine carcinomas (1). Morphologically, it shares similar features to those of neuroendocrine tumours arising from elsewhere. The diagnosis of primary neuroendocrine carcinomas of the breast can only be made if non-mammary sites are excluded and an in situ component can be found histologically (1, 2). We recently encountered an aggressive primary small cell neuroendocrine carcinoma of the breast presenting with signs of inflammatory breast disease in a 22-year-old lady. This report highlights the imaging features of this rare entity, including a Doppler ultrasonographic finding which has never been documented in previous literature.

## 2. Case Presentation

A 22-year-old nulliparous lady with no previous medical illness presented with a 6-month history of a right breast lump. The lump was described as slow growing, but rapidly enlarged 3 weeks before presentation to the hospital. She denied having nipple discharge or fever. There was no family history of breast carcinoma.

**Figure 1:**
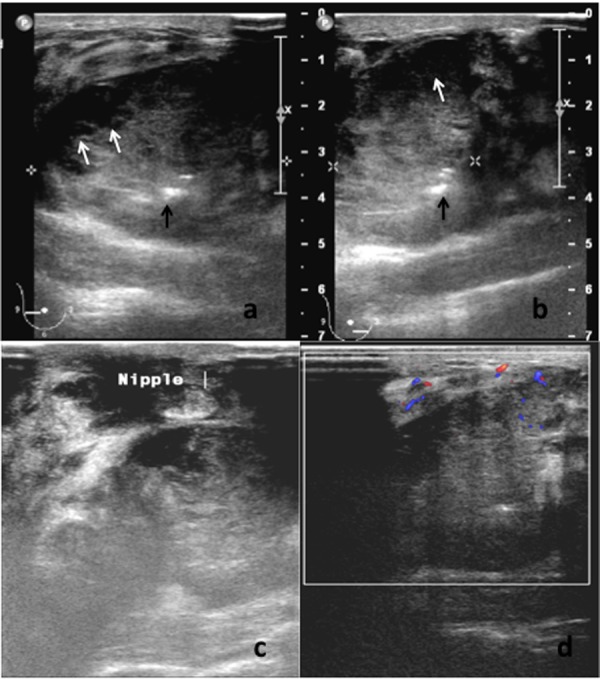
A and B, Gray-scale ultrasound examination of the right breast showing large partially delineated hypoechoic mass containing small cystic areas (white arrows) and echogenic foci (black arrows). The surrounding breast parenchyma showed heterogeneous echotexture; C, The mass is closely related to the nipple causing nipple retraction and fixation; D, Color-Doppler ultrasound study showed low intra-tumoral vascularity with moderate flow signal surrounding the mass.

On physical examination, the right breast was markedly swollen and tender. The overlying skin was warm and erythematous. A large firm mass was felt predominantly in the lower quadrants of the right breast. It was fixed to the superficial skin, nipple and underlying muscle. Several right axillary lymph nodes were palpable. The left breast and the left axilla were normal. She was afebrile with stable vital signs. Examination of the other systems was unremarkable. Blood investigation revealed normal parameters. She was immediately referred for a breast ultrasonography after a core biopsy of the mass was performed in the breast clinic. Ultrasound examination of the right breast revealed a large lobulated hypoechoic solid lesion occupying the lower quadrants and extending to the retroareolar region. The nipple was retracted towards the mass. The mass measured approximately 8.0 cm in width and 3.9 cm in height and contained cystic areas and few hyperechoic foci. The margin of the mass was partially well-delineated and mildly lobulated. Neither posterior acoustic enhancement nor shadowing was present. The surrounding breast parenchyma was heterogeneous and edematous. There was no satellite nodule. Color-Doppler study of the lesion showed minimal flow signal seen only at the medial side of the periphery of the mass. The rest of the mass was generally avascular. No penetrating vessel into the mass was seen. A moderate amount of flow signal was documented in the stroma surrounding the mass (Figure 1). Multiple enlarged right axillary lymph nodes with loss of the fatty hilum were noted with the largest measuring 1.9 × 1.2 cm. Ultrasound of the left breast and axilla was normal.

**Figure 2:**
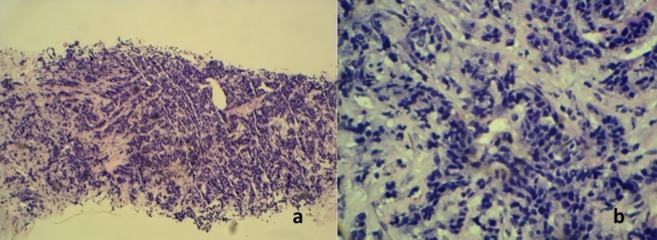
A, Histopathology of section of the tumor showing partly necrotic, focally crushed fragments of fibrofatty tissue exhibiting clusters and trabeculae of malignant epithelial cells surrounded by desmoplastic stroma (H & E stain × 10); B, The tumor cells exhibit moderately pleomorphic hyperchromatic nuclei, scanty cytoplasm and high mitotic activity (H & E stain × 40).

**Figure 3:**
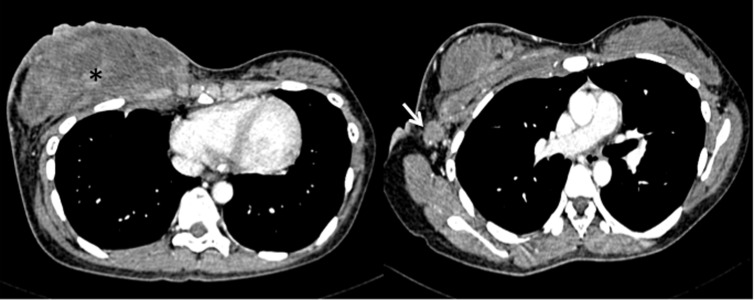
Axial enhanced CT image of the chest showing a large right breast mass (asterisks) that has infiltrated the underlying medial chest wall and a large right axillary lymph node (arrow).

Histopathological examination of the core tissue revealed partly necrotic, focally crushed fragments of fibrofatty tissue exhibiting clusters and trabeculae of malignant epithelial cells surrounded by desmoplastic stroma. There was no obvious tubule or gland formation. The tumor cells exhibit moderately pleomorphic hyperchromatic nuclei, scanty cytoplasm and a high mitotic activity (10/10 hpf) (Figure 2). Numerous apoptotic bodies were noted. Immunohisto chemically, the tumor cells were immune positive for synaptophysin, chromogranin and MNF116. Interpretation of the result was infiltrating neuroendocrine carcinoma of the breast. The patient initially refused further imaging or treatment, but returned to the clinic a month later. This time the breast mass had grown larger associated with skin ulceration. The previously palpable right axillary nodes had also become larger and fixed. A contrast enhanced computed tomography (CT) examination of the thorax, abdomen and pelvis revealed a heterogeneously enhancing mass 5.4 × 13.8 × 11.0 cm in size, occupying the whole of the right breast. The mass infiltrated the underlying pectoralis major muscle and a part of the underlying chest wall. Multiple enhancing enlarged right axillary lymph nodes were present (Figure 3). The left breast and axilla were normal. There was no significant abnormality detected in the rest of the thorax, abdomen or pelvis and including the visualized spine bones.

**Figure 4:**
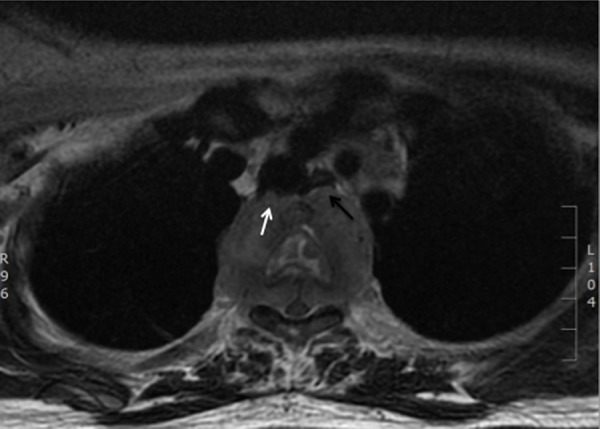
Axial T2 fat suppressed MR image at the level of T3 showing a paravertebral mass extending into the spinal canal causing spinal cord displacement and compression. The mass has also indented the posterior wall of the trachea (white arrow) and displacing the esophagus anteriorly (black arrow).

A combination of cisplatin and etoposide (100 mg/m2) neoadjuvant chemotherapy based on the protocol for small cell lung carcinoma was started immediately. Upon completion of the 4th cycle of chemotherapy, she developed bilateral lower limb weakness with urinary incontinence and sensory loss up to T4 level. Magnetic resonance imaging (MRI) of the spine (GE 1.5T) demonstrated metastases to the vertebral bodies of T2 to T4 with paraspinal and epidural soft tissue components that caused spinal canal stenosis and spinal cord compression at the T3 level. The paraspinal component was also indenting the posterior wall of the trachea and displacing the esophagus anteriorly (Figure 4). Emergency decompression laminectomy and debulking of the paraspinal tumor was performed. After the surgery, she regained sensation with slight improvement of the power of both lower limbs, but on the 9th post surgical day, she suddenly developed chest discomfort and soon went into cardio-circulatory collapse. Attempts to revive her failed. Clinically, she was suspected of having acute pulmonary embolism. Post mortem autopsy was not performed on her as family members did not agree.

## 3. Discussion

Neuroendocrine tumors are tumors composed of neuroendocrine cells that are present throughout the nervous and endocrine or hormonal systems. These include carcinoid tumor, pancreatic endocrine tumor, paraganglioma, pheochromocytoma, medullary thyroid carcinoma and poorly differentiated small cell neuroendocrine carcinoma. As neuroendocrine cells are distributed widely throughout the body, tumors of these cells can occur at many sites, but they usually occur in the lungs or gastrointestinal tracts (1, 2). The origin of neuroendocrine carcinoma in the breast is still not clear, although its histogenesis is thought to arise from multipotential stem cells capable of divergent differentiation. Primary neuroendocrine carcinoma also occurs in other sites where neuroendocrine cells are normally absent or not readily identifiable, including the ovary and prostate (3). Primary neuroendocrine carcinoma of the breast is extremely rare with the first reported case in 1983 (4). Since then, fewer than 40 cases have been reported in the literature. The most frequent reported age varies from 40 to 70 years, with a higher incidence in women greater than 60 years (3). Prior to this case, the youngest documented case was in a 31-year-old woman (2). This rare tumor has also been reported in a 52-year-old man (5). To the best of our knowledge, the presented case is the youngest reported patient of primary neuroendocrine carcinoma of the breast in the literature.

This case presented with the uncommon clinical signs of inflammatory breast involvement similarly described by Samli et al. (5). In this age group, the common inflammatory breast pathologies are cellulitis, mastitis or breast abscess. However, apart from the breast edema and skin erythema, the patient displayed no other signs of infection such as fever, chills or leukocytosis. She also did not have risk factor for mastitis such as lactation. Furthermore, the axillary lymphadenopathy, with loss of fatty hilum seen on ultrasound was highly suspicious of a malignant etiology. Reactive nodes due to infection, though enlarged, should have preservation of the fatty hilum. Among those cases reported in the literature, mammographic findings have been frequently described (3, 4, 6-13), but ultrasonographic findings have been reported in a few studies only (4, 7-11). Mammographically, this condition has been reported as a dense, lobulated mass with a partially ill-defined margin (4, 10, 11, 13). Calcification and a spiculated border are also other documented mammographic findings (8, 11). Mammography was not performed in this case because of the patient’s young age and the high reliability of ultrasound in characterizing and detecting both solid and cystic components (4). The subsequent ultrasonographic findings reported by Rubini et al. also showed a cyst-containing mass with intracystic tracts (7). However, in the more recent papers, the lesions were generally described as solid hypoechoic masses with well or ill-defined borders and microlobulations of the tumor margin (8-11). Posterior acoustic shadowing, a sonographic feature typically associated with breast carcinoma, was not documented in previous literature. On the other hand, posterior acoustic enhancement was a common feature described by few authors (9-11). Mizukami et al. explained that the posterior acoustic enhancement reflects the high cellularity of the tumor cells (10). Our ultrasound examination revealed a solid, mildly lobulated hypoechoic mass with partially well-delineated borders. The mass contained few small cystic areas and few echogenic foci. The mass exhibited normal sound transmission (no acoustic phenomena). The cystic areas were probably the result of tissue necrosis which was revealed histologically, while the internal echogenic foci most likely represent air or hemorrhagic spots following the core biopsy performed just prior to the ultrasound examination. Doppler sonography has been helpful in differentiating between benign and malignant breast lesions. The greater number of vessels and presence of penetrating vessels are characteristic patterns of vascularity that are more commonly seen in malignant lesions (14, 15). However, these malignant characteristics were not elicited in the color-Doppler study of this case. Instead, we noted poor vascularity within the tumor and absence of penetrating vessels. The flow signals seen surrounding the tumor reflects increased vascularity of the surrounding breast stroma. To date, literature on Doppler sonography of the neuroendocrine tumor of the breast is not available for comparison or review and there has been only one reported MR finding of this rare tumor in the English literature. Bilgen et al. in their retrospective review of an MR examination performed on a non-palpable histologically proven primary neuroendocrine carcinoma of the breast, described the lesion as having irregular margins and showing homogeneous contrast enhancement with a time-intensity curve that showed early enhancement suggestive of malignancy (11). Immunohisto chemically, the cells of neuroendocrine tumor will show immunore activity for specific markers of endocrine differentiation. However, there is no consistent pattern of neuroendocrine marker expression in primary neuroendocrine breast carcinoma (2). In this case, the tumor showed a positive reaction to synaptophysin, chromogranin and cytokeratin MNF116. It also showed a high nuclear-to-cytoplasmic ratio, hyperchromatic nuclei and a high mitotic rate. Histologically, it is not possible to distinguish a metastatic and primary neuroendocrine tumor of the breast (6). The diagnosis of primary breast tumor is made on the basis of radiologic findings that exclude other non-mammary tumors and presence of an in situ component demonstrated within the breast histopathologically (2, 10). Generally, this tumor is considered clinically aggressive with dismal prognosis. As observed in this case, previous authors have also reported rapid progression or recurrence of this disease locally and distally, and a short survival period (1, 4, 6, 15). However, as in breast carcinoma of the usual type, size is a very important prognostic factor for this tumor (3). In more recent reports, it seems that the prognosis is better if the tumors are detected early and if there is no lymph node metastasis (3, 8). As for the management, there is still no established standard treatment protocol because so few cases have been described. As this entity resembles small cell lung carcinoma in morphology, clinical behavior and histiogenesis, it seems reasonable that their treatment should be similar (2). Thus, our patient was given chemotherapy using protocol for small cell lung carcinoma. Surgical excision may be implemented if the tumor responds well to chemotherapy and becomes operable. Unfortunately, in the presented case, chemotherapy failed to control the tumor and the disease followed a rapid and fatal course. The prognosis in this case is governed by the clinical stage of the disease at presentation.

In conclusion, primary neuroendocrine carcinoma of the breast is a rare entity. Reviewing the limited literature on this tumor, there is no age limit, classical presentation or specific imaging feature. It can occur in a 22-year-old young lady or even mimic an inflammatory breast condition. It displays some, but not the entire characteristic ultrasonographic features of a malignant breast lesion. Awareness of this type of breast tumor may help in the differential diagnosis. Although primary neuroendocrine carcinoma of the breast is regarded as clinically aggressive with poor outcome, early detection may lead to a more favorable prognosis.
